# Coal consumption and carbon emission reductions in BRICS countries

**DOI:** 10.1371/journal.pone.0300676

**Published:** 2024-03-29

**Authors:** Jie Wen, Fan Yang, Yiyin Xu

**Affiliations:** 1 School of Public Finance and Taxation, Southwestern University of Finance and Economics, Chengdu, China; 2 School of Public Administration, Southwestern University of Finance and Economics, Chengdu, China; 3 Business School, Chengdu University, Chengdu, China; Soochow University, CHINA

## Abstract

The primary energy consumption structure of BRICS countries is dominated by fossil energy, particularly coal. Coal consumption in BRICS countries is a major driver underlying increased carbon emissions. Therefore, this study developed a spatiotemporal decoupling mode and incorporated factors related to coal consumption-induced carbon emissions into a spatiotemporal decoupling analysis method to provide differentiated and targeted policies for energy restructuring and emission reduction targets in BRICS countries. Moreover, a temporal-spatial decomposition logarithmic mean Divisia index model was developed using the spatiotemporal decoupling index method. The model is based on CO_2_ emissions generated by coal consumption in BRICS countries, with a primary focus on data from Brazil, Russia, South Africa, India, and China. The findings reveal distinct spatiotemporal distributions and driving effects of coal consumption and carbon dioxide emissions across various countries. Factors such as CO_2_ emission intensity, coal consumption intensity, economic output per capita, and population structure exerted either positive or negative effects on the distributional effect of the carbon emission-economic output per capita association in BRICS countries. Additionally, country-level heterogeneity in the influence of the distributional effects of CO_2_ emissions was observed within each BRICS country. Thus, different policies are needed to achieve carbon emission reduction targets in different countries.

## 1. Introduction

Climate change is a major global challenge. Large-scale greenhouse gas emissions have led to increasingly severe greenhouse gas effects, which have exacerbated extreme climate change and highlighted the increasing prevalence of global environmental problems [1]. CO_2_ is a major greenhouse gas that has been consistent focus of the international community [2]. In recent years, BRICS countries have become important drivers of global economic development as well as major contributors to global CO_2_ emission growth; thus, carbon emissions from BRICS countries are facing more stringent reduction emission targets under the Paris Agreement [3]. In recent decades, BRICS countries, which account for 41% of the global population, have faced rapid urbanization along with economic development and industrialization, which have led to associated increases in carbon emissions. Although BRICS countries represent the fastest-growing countries in the world, they are also the main contributors to carbon emissions. Total BRICS-associated CO_2_ emissions account for 45.8% of the global carbon emissions.

**Fig 1:**
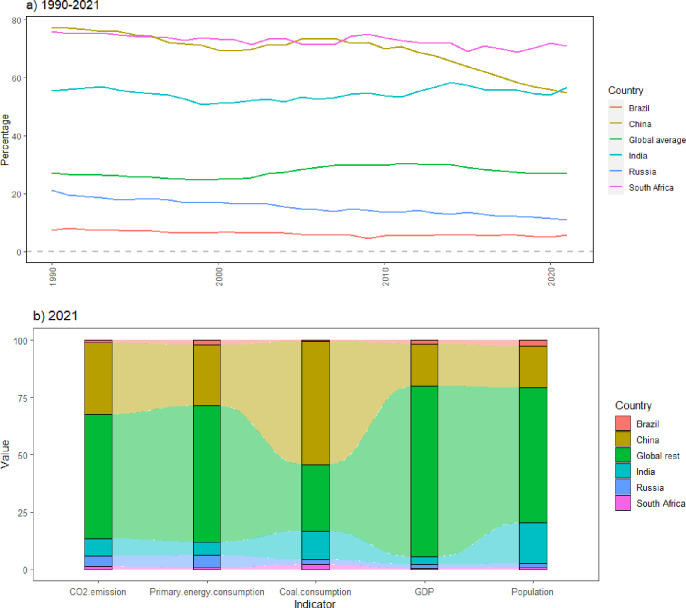
Coal and primary energy consumption structure and the carbon emissions share of BRICS (1990 to 2021). Note: a) Coal-primary energy consumption structure of BRICS countries. b) CO_2_ emissions share of BRICS countries.

Moreover, influenced by differences in economic development, technology, and energy endowment, BRICS countries present primary energy consumption structures that are still dominated by fossil energy, among which coal is the main source. Among BRICS countries, China and India are the first and second largest global coal consumers, respectively, and the coal consumption of the five BRICS countries Brazil, Russia, South Africa, India, and China accounts for 71% of the worldwide total (In 2021, coal consumption in BRICS countries accounted for nearly three-quarters (71.15%) of the global total coal consumption.). Studies have shown that coal is the principal source in energy-related carbon emissions [4]. The larger the share of coal in a nation’s energy structure, the higher the intensity of its CO_2_ emissions [5]. In other words, coal consumption in BRICS countries is the major driver of carbon emission growth. Fig 1 shows the share of coal consumption in primary energy in BRICS and reveals that it is much higher than the global level except in Brazil and Russia.

Considering the increasing demand for energy due to economic growth, population size, industrialization, and urbanization, changing the existing energy consumption structure over long development period is unrealistic in BRICS countries. Because the marginal cost of maintaining coal energy is lower than that of exploiting and using renewable and sustainable energy resources and current technological advances have lowered the production and operation costs of traditional fossil energy sources and increased the energy efficiency, coal will continue to be consumed for a long time as BRICS countries attempt to realize benefits and cost-effectiveness during economic development [6, 7]. This trend has also been reinforced based on turbulent global political situations. The global energy crisis caused by the Russia-Ukraine military conflict that began in February 2022 prompted Europe to return to a coal-based power generation energy consumption structure [8]. Therefore, the issue of coal consumption and carbon emissions in BRICS countries must be further discussed to realize global CO_2_ emission reductions.

## 2. Literature review

BRICS countries have been studied from multiple perspectives. Some studies have explored the impact of foreign direct investment (FDI) on the technological innovation in BRICS countries [9], and the impact of FDI and green innovation on the quality of the economic environment in BRICS countries [10]. Other studies discuss the impact of technological innovation and natural resources in energy-growth-environment nexus in BRICS economies [11] and the effects of carbon emissions, renewable energy consumption, FDI, and exports on economic growth in BRICS countries [12]. A recent study applied hidden cointegration and asymmetric causality tests to estimate the asymmetric nexus between energy growth and CO_2_ emissions for BRICS countries [13]. Most existing literature on BRICS focuses on the relation between economic growth, urbanization, energy resources consumption, and CO_2_ emissions of BRICS countries [14–18], while a few studies focus on the connection between renewable energy development and carbon emissions in BRICS [19–23]. Only several studies have conducted exponential decomposing of the factors influencing carbon emissions in BRICS countries, but they are limited to temporal decomposition over a certain time span [24–26] with remarkably different results. For instance, de Freitas and Kaneko argued that economic activities and population are the major contributors to the increase in CO_2_ emissions in Brazil, and energy intensity effect is not significant. According to Dai et al., the energy intensity effect has had a positive impact on carbon emission reductions in all BRICS countries. Inglesi-Lotz argued that increasing energy intensity in South Africa has led to an increase in carbon emissions, thus demonstrating the role of the energy rebound theory. However, spatial decomposition and decoupling analyses of the differences among BRICS countries and the influencing factors have not been previously performed. This study suggests that the ideal situation for maintaining a sufficient growth rate in BRICS countries is to balance the use of coal while reducing CO_2_ emissions or ensure that the decrease in the share of coal consumption is higher than the increase in CO_2_ emissions while maintaining a high rate of economic growth, urbanization, and industrialization. Such factors are appropriate for a decoupling analysis. Chen et al. [27] indicated that the concept of "decoupling" comes from physics and defines a state in which the relation among relevant physical quantities is weakened or absent. Such "decoupling" analyses have been applied in a wide range of domains, particularly in terms of carbon emissions and economic growth [28].

The Kaya constant equation is a model for explaining the relationship between CO_2_ emissions and socio-economic variables, and it has become one of the main approaches for CO_2_ emissions research. This model may be used in both discrete and continuous time settings. A typical practice is to use the Kaya identity as the scheme and the Divisia index as the decomposition technique [29–31]. Utilizing the Kaya identity, Raupach et al. [32] employed worldwide data obtained from the U.S. Energy Information Administration (EIA) and the U.S. Department of Energy’s Carbon Dioxide Information and Analysis Center (CDIAC). They integrated this information with six CO_2_ emission scenarios set by the Intergovernmental Panel on Climate Change (IPCC). The purpose was to demonstrate the causes of global CO_2_ emission changes, and the reasons for the change of major carbon dioxide emission entities. Subsequent studies have focused on this framework [33, 34].

Another interesting theme is related to the type of energy source used. Coal is the principal single source of energy-related carbon emissions and a major contributor to climate change and environmental degradation [35]. Its use for production and consumption causes severe environmental and socioeconomic problems and has a significant influence on global carbon emissions and sustainable development. While the need for socioeconomic development remains a key consideration in the national strategies of many countries, global warming and climatic change have caused the international community to focus on the effects of coal consumption. Several studies on coal consumption in BRICS countries as a whole or in a particular country have been conducted [36–41] (Chen et al., 2018; Adedoyin et al., 2020; Chandran Govindaraju and Tang, 2013; Aleksandrovna Rodionova et al., 2017; Li and Li, 2011; Bloch et al., 2012; Shahbaz et al., 2013). For example, Chen et al. provided an in-depth discussion of coal consumption and CO_2_ emission reductions in the global top three coal consumers and CO_2_ emitters (China, USA, and India). However, the CO_2_ emission data used in these studies were based on energy consumption rather than coal consumption only.

The above literature shows that although studies have investigated the relationship between economic growth, energy sources, and carbon emissions in BRICS countries, the connection between coal energy and carbon emissions generated by coal consumption has rarely been studied. Therefore, this study contributes to the existing literature by filling this gap.

First, we decompose the CO_2_ emission changes caused by coal consumption in the five BRICS countries and associated factors and analyze the motivations and mechanisms behind these factors, which are important to identify so that BRICS countries can formulate corresponding policies. These countries are at different stages and under different pressures because they vary in many aspects, such as economic development and natural resource endowment. Identifying CO_2_ emissions from coal and its factors can help each country formulate its own coal consumption policies and make relevant adjustments to promote CO_2_ emission reductions.

Second, a spatial-temporal decomposition approach is used as a study method to examine the drivers of CO_2_ emission differences caused by coal consumption among BRICS countries and country clusters. Through spatial logarithmic mean Divisia index (LMDI) decomposition, the study discusses the driving factors of inter-regional CO_2_ emission differences to provide differentiated and targeted policies for energy restructuring and emission reduction targets in BRICS countries.

Third, a newly developed spatial decoupling analysis is applied to the CO_2_ emissions of BRICS countries by incorporating the effects of economic activities and population growth. Based on the spatial analysis, the changes and drivers in the spatial distribution effects of carbon emissions from coal in different nations can be comprehensively investigated, thereby filling the gap in the existing literature.

## 3. Research methods and data

### 3.1 Temporal decomposition of the drivers of CO_2_ emissions changes

With the best numerical properties and economic implications, LMDI is a good factor decomposition analysis with appropriate statistical properties [42, 43]. Based on Kaya’s constant equation and the LMDI approach, the paper integrates the effects of carbon emissions intensity, energy consumption intensity, economic output, demographic structure, and population size on carbon emission changes in BRICS countries as follows.


$$C_{coal}=\sum_{i}\frac{C_{coal,i}}{{COE}_{i}}∙\frac{{COE}_{i}}{Y_{i}}∙\frac{Y_{i}}{P_{i}}∙\frac{P_{i}}{P}∙P=\sum_{i}CI_{i}∙EI_{i}∙y_{i}∙PS_{i}∙P$$ (1)

Where C_coal_ is the carbon emission from coal consumption, COE is coal consumption, Y denotes GDP, and P denotes population size. The definition of ${CI}_{i}=\frac{C_{coal,i}}{{COE}_{i}}$ is carbon emission intensity from coal consumption, ${EI}_{i}=\frac{{COE}_{i}}{Y_{i}}$ is coal consumption intensity, $y_{i}=\frac{Y_{i}}{P_{i}}$ is GDP per capita, ${PS}_{i}=\frac{P_{i}}{P}$ is population structure, and i refers to the ith country.

Furthermore, the CO_2_ emissions changes from coal consumption can be described as follows.


$$\Delta C_{coal}=C_{coal}^{t}-C_{coal}^{b}=\Delta C_{CI}+\Delta C_{EI}+{\Delta C}_{y}+\Delta C_{PS}+\Delta C_{P}$$ (2)

Where ΔC_coal_, ΔC_CI_, ΔC_EI_, ΔC_y_, ΔC_PS_, ΔC_P_ is the total effect, CO_2_ emission intensity effect, coal consumption intensity effect, economic output per capita effect, population structure effect, and population size effect of carbon emission from coal consumption respectively.

If ΔC_CI_, ΔC_EI_, ΔC_y_, ΔC_PS_, and ΔC_P_ change in the same direction of ΔC_coal_ change, then these factors will be seen as the drivers of carbon emission growth. Otherwise, they reduce carbon emissions.

### 3.2 Spatial LMDI decomposition

In this study, the spatial decomposition method of Cheng et al. [44] was used with the average of BRICS countries as a benchmark for comparing and ranking the average of the five countries Brazil, Russia, South Africa, India, and China to identify regional differences in carbon emissions. The difference in ${\Delta C}_{i}^{MR}$ in terms of carbon emissions between country i and the average of the five countries is expressed by Eq (3).

$${\Delta C}_{i}^{MR}=C_{i}-C^{*}$$ (3)

where C* is the mean value in carbon emission from coal consumption in BRICS. Accordingly, the difference in terms of the average carbon emissions between country i and the mean value of the five countries can be determined using Eq (4):

$${\Delta C}_{i}^{MR}={\Delta C}_{i,CI}^{MR}+{\Delta C}_{i,EI}^{MR}+{\Delta C}_{i,y}^{MR}+{\Delta C}_{i,PS}^{MR}+{\Delta C}_{i,P}^{MR}$$ (4)

Considering the different economic development levels, population sizes, and carbon emissions in BRICS, this paper divides the five countries into two groups. China and India rank as the first and second largest countries in terms of global population as well as consumers of coal consumption, respectively, and are divided into one group, while Brazil, Russia, and South Africa are divided into the other. The differences in carbon emissions between each country and the mean value of the five countries are decomposed into two components. The first component is the difference between the carbon emission of each country and the mean value in carbon emission of its country cluster (intra-spatial LMDI decomposition). The second component is the difference between the average carbon emissions of each country cluster and the average emissions of the five countries (inter-spatial LMDI decomposition). The former discloses the driving factors of differences in carbon emissions for countries with similar energy consumption and population growth, whereas the latter shows the factors of differences in carbon emissions across country clusters with different energy consumption and population growth. This approach is expressed by Eq (5).

$${\Delta C}_{i}^{MR}=C_{i}-C^{*}=(C_{i}-C_{i}^{*})+(C_{i}^{*}-C^{*})={\Delta C}_{i}^{MR-within}+{\Delta C}_{i}^{MR-between}$$ (5)

where C_i_* is the average carbon emissions of the country cluster, C_i_^MR-within^ is the difference between the carbon emissions of country i and the average carbon emissions of the country cluster, and C_i_^MR-between^ is the difference between the average carbon emissions of the country cluster and that of the five countries.

### 3.3 Tapio elasticity decomposition and spatial decoupling decomposition

This study further integrates the LMDI and Tapio decoupling models to explore the decoupling relation between carbon emissions and economic output per capita in each country. On the basis of the Tapio model [45], the decoupling elasticity index of carbon emissions and economic output per capita can be written as follows:

$$D_{{CO}_{2},y}^{b,t}=\frac{{\Delta C}_{coal}^{b,t}/C_{coal}^{b}}{{\Delta C}_{y}^{b,t}/C_{y}^{b}}$$ (6)

Substituting Eq (2) into Eq (6) and rearranging the terms yields the following:

$$D_{{CO}_{2,y}}^{b,t}=\frac{C_{y}^{b}}{{∆C}_{y}^{b,t}}∙\frac{1}{C_{coal}^{b}}∙({∆C}_{CI}^{b,t}+{∆C}_{EI}^{b,t}+{∆C}_{y}^{b,t}+{∆C}_{PS}^{b,t}+{∆C}_{P}^{b,t})=\underset{Interactive-effect}{\underset{︸}{\frac{C_{y}^{b}}{{∆C}_{y}^{b,t}}∙\frac{1}{C_{coal}^{b}}∙({∆C}_{CI}^{b,t}+{∆C}_{EI}^{b,t}+{∆C}_{PS}^{b,t}+{∆C}_{P}^{b,t})}}+\underset{Direct-effect}{\underset{︸}{\frac{C_{y}^{b}}{C_{coal}^{b}}}}$$ (7)

To determine the determinants of the spatial distributional effect of carbon emission, we introduce the spatial decoupling index decomposition approaches of Chen et al. [46]. The CO_2_ emissions of Country i were modeled as follows:

$$C_{coal,i}=\frac{C_{coal,i}}{{COE}_{i}}∙\frac{{COE}_{i}}{Y_{i}}∙\frac{Y_{i}}{P_{i}}∙\frac{P_{i}}{P}∙P=CI_{i}∙EI_{i}∙y_{i}∙PS_{i}∙P$$ (8A)

Where C_coal_ is the CO_2_ emission from coal consumption, COE is coal energy consumption, Y denotes GDP, and P denotes population size. The definition of ${CI}_{i}=\frac{C_{coal,i}}{{COE}_{i}}$ is the CO_2_ emissions intensity from coal consumption, ${EI}_{i}=\frac{{COE}_{i}}{Y_{i}}$ is the intensity of coal consumption, $y_{i}=\frac{Y_{i}}{P_{i}}$ is per capita GDP, ${PS}_{i}=\frac{P_{i}}{P}$ is population structure, and i refers to the ith country.

Similarly, the average CO_2_ emission for each group j can be described as follows:

$$C_{coal,j}=\frac{C_{coal,j}}{{COE}_{j}}∙\frac{{COE}_{j}}{Y_{j}}∙\frac{Y_{j}}{P_{j}}∙\frac{P_{j}}{P}∙P=CI_{j}∙EI_{j}∙y_{j}∙PS_{j}∙P$$ (8B)

The absolute difference in CO_2_ emissions between country i and group j can be completely decomposed as follows:

$${\Delta C}_{coal-i,j}=\Delta {CI}_{i,j}+\Delta {EI}_{i,j}+{\Delta y}_{i,j}+\Delta {PS}_{i,j}+\Delta P_{i,j}$$ (9)

Where *ΔCI*_*i*,*j*_, ΔEI_i,j_, Δy_i,j_,ΔPS_i,j_, and ΔP_i,j_ denote the CO_2_ emission intensity effect, coal consumption intensity effect, per capita GDP effect, population structure effect, and population size effect of the absolute difference between BRICS country i and its group j respectively.

Finally, the spatial decoupling index can be further decomposed as follows:

Di,j=∆Ccoal−i,jCcoal,j∆yi,jyj=(∆CIi,j+∆EIi,j+∆yi,j+∆PSi,j+∆Pi,j)Ccoal,j∆yi,jyj=yj∆yi,j∙Ccoal,j∙∆CIi,j+yj∆yi,j∙Ccoal,j∙∆EIi,j+yj∆yi,j∙Ccoal,j∙∆yi,j+yj∆yi,j∙Ccoal,j∙∆PSi,j+yj∆yi,j∙Ccoal,j∙∆Pi,j=Di,jCI+Di,jEI+Di,jy+Di,jPS+Di,jP (10)

In Eq (10), the overall spatial decoupling index D_i,j_ is decomposed into five sub-indices (denoted as $D_{i,j}^{sub}$). $D_{i,j}^{sub}$ includes $D_{i,j}^{CI},\;D_{i,j}^{EI},\;D_{i,j}^{y},\;D_{i,j}^{PS},\;\mathrm{and}\;D_{i,j}^{P}$, which represent the effects of carbon emission intensity, coal consumption intensity, GDP per capita, population structure, and population size on carbon emission changes, respectively.

Specifically, when Δy_i,j_ > 0 and $D_{i,j}^{sub}$ < 0, then the distributional effect of the relation between carbon emissions and economic output per capita is positive; whereas when Δy_i,j_ > 0 and $D_{i,j}^{sub}$ > 0, then the distributional effect is negative. Similarly, when Δy_i,j_ < 0 and $D_{i,j}^{sub}$ > 0, then the distributional effect is positive; whereas when Δy_i,j_ < 0 and $D_{i,j}^{sub}$ < 0, then the distributional effect of the relation between carbon emissions and economic output per capita is negative.

### 3.4 Data

Based on data availability and a consistent statistical scale, this study mainly focuses on data from the five BRICS countries of Brazil, Russia, South Africa, India, and China from 2010 to 2018. Data on CO_2_ emissions generated by coal were obtained from the *Annual Report 2021 for Carbon Dioxide Emission Accounts of Global Emerging Economies* [47], which was published in November 2021 by Carbon Emission Accounts and Datasets (CEADs). Other energy data were obtained from the *BP Statistical Review of World Energy—2022*, *71st edition* (BP, 2022), and data on population size, GDP and GDP per capita were mainly from the UN and World Bank websites.

## 4. Empirical analysis

### 4.1 Temporal LMDI decomposition and decoupling analysis

**Fig 2:**
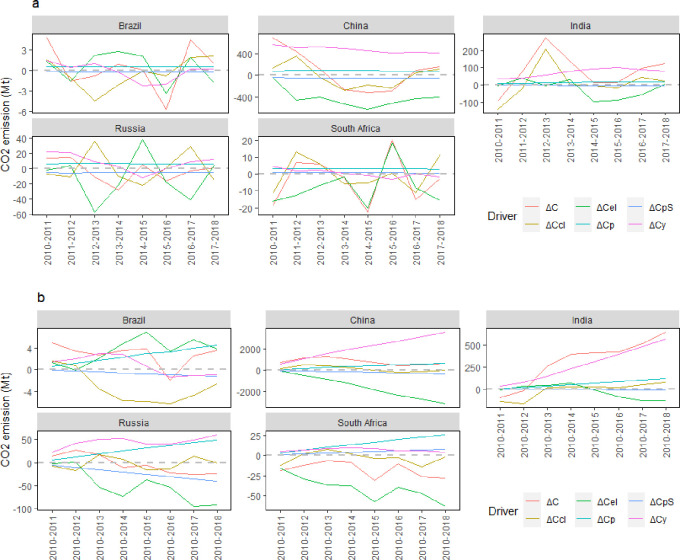
Decomposition of the drivers of carbon emissions from coal in BRICS countries (year-by-year effects and cumulative annual effects). Note: a) stands for year-by-year effects and b) stands for cumulative annual effects.

This study employed the LMDI approach to decompose the driving factors of carbon emissions in BRICS countries. The results are shown in Fig 2.

Brazil’s CO_2_ emissions generated by coal consumption changed moderately. Despite the significant decline in 2016, the emissions quickly rose to the average level with a moderate overall trend, which coincides with the data on coal consumption in Brazil. The emissions in 2016 had a value of 0.667 EJ, which was 9.67% less than that in 2015, and then rose rapidly in 2021 to 0.712 EJ, showing a more stable trend in coal consumption overall. This is because Brazil’s fossil fuel consumption is dominated by petroleum products, which is the most essential contributor to carbon emissions generated by fossil energy in Brazil.

Fig 2 reveals the decomposition results of the drivers of carbon emissions from coal energy consumption in BRICS. The intensity of CO_2_ emissions and coal consumption are the most important drivers of carbon emission changes in Brazil, and they have opposite influences, with the former curbing CO_2_ emissions and the latter boosting CO_2_ emissions. Since coal is the only energy source considered in this study, this finding indicates that Brazil has failed to make effective progress in coal energy technology. The economic output per capita effect promoted CO_2_ emissions before 2015 and gradually acted as a damper after 2016, which is consistent with existing studies, suggesting that Brazil’s economic growth is not mainly based on energy consumption. A possible reason for this might be that Brazil has rich agricultural resources and relatively slow industrialization, of which agriculture is the mainstay of its economy. The population size effect increases CO_2_ emissions, reflecting that population growth expands the scale of energy development and utilization, which results in an increase in carbon emissions. This demographic effect reduces CO_2_ emissions, thus reflecting a continuous decrease in the population ratio in Brazil.

China’s carbon emissions from coal consumption fluctuated in recent years, reaching a very high value of 7,668.09 Mt in 2013, decreased over the next few years, and then increased in 2017, reaching 7,041.99 Mt in 2018. China’s fossil energy consumption is still dominated by coal; however, the proportion has been decreasing gradually from 70.01% in 2010 to 58.36% in 2018, indicating that China is accelerating the pace of coal reduction and strictly controlling coal consumption. China’s CO_2_ emissions have been increasing but at a slower rate than before, while the share of CO_2_ emissions from coal consumption decreased from 78.39% in 2010 to 72.95% in 2018. This indicates that China’s CO_2_ from coal consumption is the major contributor to CO_2_ emission growth, and that China’s economic growth is gradually reducing its dependence on coal consumption.

Fig 2 shows that the effects of per capita economic output and population size in China present the same change trends as carbon emissions, while the effects of carbon emission intensity, coal consumption intensity, and population structure present opposite change trends as CO_2_ emissions. Among the five effects, economic output per capita and coal consumption intensity are the most important drivers that increase and reduce CO_2_ emissions in China, respectively. China is likely to face more pressure to reduce CO_2_ emissions in the future as long as its economy continues to grow. Fortunately, energy consumption intensity offsets the effect of per capita economic output. Therefore, if China’s coal consumption efficiency continues to improve, supported by a package of measures, such as internationally advanced coal power technology, China still has the opportunity to achieve its CO_2_ reduction target. The effects of the population structure, population size, and CO_2_ emission intensity were relatively small. Nonetheless, their significance in China’s future CO_2_ emission reduction cannot be ignored. For instance, compared to 2010, the effect of coal consumption intensity decreased by 4,855 million tons in 2018, the effect of economic output per capita increased by 5,254 million tons, the population structure effect decreased by 347 million tons, the population size effect increased by 629 million tons, and the CO_2_ emission intensity effect decreased by 0.26 million tons. This indicates that after the effects of economic output per capita offset the effects of energy consumption intensity, population structure, population size, and CO_2_ emission intensity determine the CO_2_ emission trends in China, of which China’s population structure is influenced by the domestic fertility policy. In recent years, the impact of the one-child policy, which has been implemented for many years, has gradually emerged, and combined with the continuous improvement in China’s social welfare, China’s low fertility rate and aging population are becoming increasingly serious issues. Therefore, the effect of population structure might skyrocket, and decreasing population growth will reduce China’s CO_2_ emissions in the future. This is also clearly shown in Fig 2, where the effect of the population structure makes a decreasing contribution to CO_2_ emissions. The effect of population size increases CO_2_ emissions, which is in accord with the findings in Brazil. The effect of CO_2_ emission intensity reduces CO_2_ emissions. In addition to the effect of population structure, only CO_2_ emission intensity can offset future CO_2_ emissions caused by per capita economic output. In this study, CO_2_ emission intensity reflects the coal consumption structure. To achieve future CO_2_ emission reduction targets, China must continue to reduce its coal consumption.

India’s CO_2_ emissions from coal consumption increased during the reporting period. As the third-largest energy sources consumer and carbon dioxide emitter worldwide, India’s fossil energy consumption remains dominated by coal; thus, carbon emissions from fossil energy are primarily accounted for by coal. CO_2_ emissions in India increased significantly from 1,040.01 Mt in 2010 to 1,687.88 Mt in 2018. It is predicted that the increase of coal consumption in India will last for quite a long period, and its CO_2_ emissions will also grow steadily. Therefore, India’s CO_2_ emissions should receive increased attention.

In addition to the energy consumption intensity and population structure, India’s per capita economic output, population size, and carbon emission intensity have all increased its’ CO_2_ emissions. Among them, per capita economic output is the largest driving factor of India’s CO_2_ emissions increase, implying that India’s economic growth is still dependent on large-scale energy consumption, especially coal consumption, and its economic growth still comes at the cost of carbon dioxide emissions increases. India has historically relied on fossil energy for electricity production, especially coal consumption (Chen et al., 2018). Therefore, the larger India’s share of the global economy, the greater the CO_2_ emissions, which is another important reason for focusing on reducing India’s CO_2_ emissions.

Compared with China, the effect of energy intensity on carbon emission change is more significant in India. China’s energy intensity has been the most principal force in the decrease in CO_2_ emissions, while for India, the impact fluctuates. India’s energy consumption intensity increased CO_2_ emissions from 2011 to 2014, reduced CO_2_ emissions after 2014, and had a weak suppression effect from 2017. This suggests that India’s economic development is not as good as expected and that energy efficiency and energy consumption technologies need to be improved. Therefore, India must work on improving its energy efficiency and energy-saving technology across all sectors of the economy. In contrast, while demographic effects also restrain the increase in CO_2_ emissions, their impact is much smaller than that of energy consumption intensity. India’s CO_2_ emission intensity has stimulated an increase in CO_2_ emissions, and this relationship has been particularly evident since 2016. That is, CO_2_ emission intensity is gradually becoming a driver of accelerating CO_2_ emissions in India, indicating that India’s energy structure is not moving in a low-carbon direction but rather is characterized by high carbon content. Because India is a coal-rich country with high coal production and relatively low cost of using coal, there is more incentive to increase coal consumption to replace oil and other energy consumption [48].

The overall CO_2_ emissions from coal consumption in Russia have been decreasing annually, with a maximum of 561.68 Mt reached in 2012. The reason lies in the fact that Russia’s fossil energy consumption is dominated by natural gas, with coal accounting for a low share. Because of the high-carbon nature of coal, both coal and natural gas consumption are the most important contributors to CO_2_ emissions from fossil fuels in Russia; however, the increase in natural gas consumption led to more gas-related CO_2_ emissions than coal-related emissions after 2010. Generally, overall CO_2_ emissions from coal consumption in Russia are decreasing.

As illustrated in Fig 2, the effects of per capita economic output and coal consumption intensity are the most important drivers of carbon emission changes in Russia, and they affect CO_2_ emissions in opposite directions, with the former increasing CO_2_ emissions and the latter depressing CO_2_ emissions. The continued increase in economic output leads directly to CO_2_ emissions, implying that Russia’s economic growth remains dependent on large-scale energy consumption. With abundant natural gas and oil reserves, Russia associates the costs and benefits of energy production with economic viability rather than supporting renewable energy alternatives. The effect of energy consumption intensity suggests that Russia is promoting more energy-efficient coal-use technologies to reduce emissions reductions [49]. The effect of CO_2_ emission intensity fluctuates, coinciding with the increase and decrease in coal consumption in Russia during this period. The effect of population size increases CO_2_ emissions, which is consistent with the countries mentioned above. The effect of the population structure dampens CO_2_ emissions, similar to that in Brazil.

Carbon emissions from coal energy consumption in South Africa have declined rapidly and have largely declined since 2014 (except in 2016). South Africa has abundant coal reserves, and its fossil energy consumption is still dominated by coal. Among the CO_2_ emissions from fossil consumption, the consumption of coal and petroleum products is the principal source of CO_2_ emissions from fossil fuels consumption in South Africa, accounting for 71.52%. Nonetheless, from 2010 to 2018, carbon emissions from coal consumption in South Africa decreased by 9.89%, from 288.41 Mt to 259.88 Mt.

Fig 2 illustrates the results of the decomposition of the drivers of CO_2_ emissions from coal consumption in South Africa. Among them, the effects of coal consumption intensity, population structure, and population size are in the same direction as that of carbon emissions, and the effect of economic output per capita is in the opposite direction to that of carbon emissions; carbon emission intensity increases carbon dioxide emissions in the first four years, but reduces emissions in the second four years. Among the five effects, economic output per capita and energy consumption intensity are the most principal drivers underlying increases or decreases in CO_2_ emissions in South Africa, respectively, which is completely different from the dynamics in China. The effect of energy consumption intensity reflects the level of energy use efficiency and energy-efficient technologies. With coal as its main primary energy, South Africa’s coal utilization efficiency and energy consumption technologies need to be improved. Economic output per capita reflects the level of national production and economic development per capita. South Africa has experienced economic weakness and social turbulence in recent years, with its GDP per capita falling to a historical low of US$5,272 in 2016 [50]; therefore, the economic downturn has led to a reduction in South Africa’s CO_2_ emissions. Although CO_2_ emission intensity, population size, and population structure play a smaller role, their significance for the future reduction of CO_2_ emissions in South Africa cannot be ignored. Because CO_2_ emission intensity reflects the energy consumption mix, a change in the mix indicates that although South Africa is still dominated by coal consumption, it is making efforts to adjust its energy mix and seek energy transition to diversify its energy sources and has achieved some success [51]. The effect of population size increases carbon emissions, which is in accord with other countries. By contrast, although the effect of population structure also increases CO_2_ emissions, it plays a smaller role than the population size effect.

**Fig 3:**
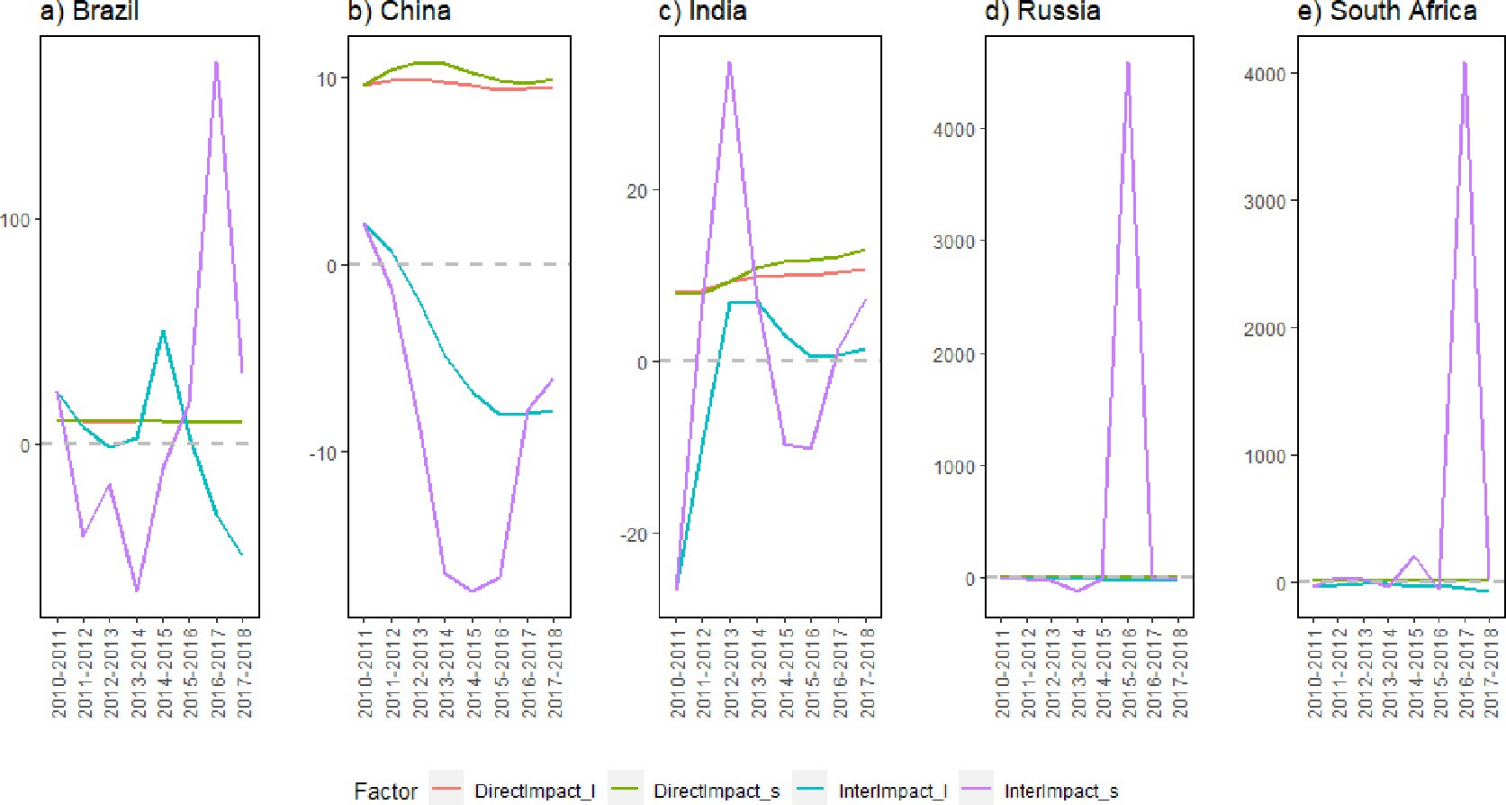
Decomposition of the elasticity of decoupling CO_2_ emissions and GDP per capita in BRICS countries: Direct and interactive effects.

Economic output per capita is a positive contributor to CO_2_ emission changes [52]. BRICS countries are all emerging or developing economies, and considering their fast-growing economies and large populations, economic output per capita can be a better indicator. Therefore, we calculated the decoupling elasticity values of CO_2_ emission changes and economic output per capita for the five BRICS countries from 2010 to 2018 and discussed the decoupling relation between CO_2_ emissions and economic output per capita, as illustrated in Fig 3.

Fig 3 shows the decoupling elasticity index between carbon emissions and economic output per capita in BRICS countries and the effects of CO_2_ emission intensity, energy consumption intensity, per capita GDP, population structure, and population size on the decoupling elasticity. In terms of year-by-year effects, the elasticity index value of Brazil fluctuated and showed a trend from expansive negative decoupling (END), strong decoupling (SD), strong negative decoupling (SND), and recessive decoupling (RD) to END from 2011 to 2018. From the perspective of cumulative annual effects, decoupling changes from END to SND were observed. While CO_2_ emissions from coal consumption have increased, the economic output has decreased. Although the decoupled state shows a trend of decoupling economic growth from carbon emissions, Brazil’s economy shows a downward trend.

The year-by-year effects of the elasticity index of CO_2_ emissions and per capita economic output in China changed from END to SD until 2016. This indicates that coal carbon emissions increase with an increase in economic output. Nevertheless, the rate of increase in economic output was greater than that of carbon emissions and decoupling was gradually achieved, indicating that the dependence of economic development on coal energy consumption gradually decreases. In terms of cumulative annual effects, END and expansive coupling (EC) (from 2016 to 2018) between economic output per capita and CO_2_ emissions were maintained, indicating that CO_2_ emissions from coal consumption increased with increasing economic output. Nonetheless, the basic situation was that CO_2_ emissions increased faster than economic output, reflecting that CO_2_ emissions were still strongly influenced by per capita economic output. Economic development and carbon emission were not decoupled during this period, although a weakening trend was observed. This is consistent with the above discussion. When the share of coal in China’s energy mix continues to decreased or the share of non-fossil fuels in primary energy increases with decreasing dependence of economic growth on coal consumption, the growth of China’s carbon emissions will continue to slow and eventually achieve a reduction in the overall scale of carbon emissions.

The elasticity index of CO_2_ emissions and economic output per capita in India corresponds to END with SD from 2011 to 2012 in terms of both year-by-year and cumulative annual effects. This indicates that coal emissions increased with increasing economic output, although the rate of increase in economic output was smaller than that of carbon emissions, reflecting that India’s economic growth during this period not only relied on the increase in coal energy consumption but also on the slight improvement in energy use efficiency and energy consumption technology, with a weak reduction in carbon emissions. This finding is consistent with the results of the previous analysis. If India cannot change its energy mix, which included a high proportion of coal consumption, and improve the quality of economic development, then its carbon emissions will not be effectively curbed.

The value of the elasticity index fluctuated greatly in terms of the year-to-year effect in Russia, showing a trend from END, SD, SND, and RD to EC; however, from the perspective of the cumulative annual effect, it has remained in a state of SD since 2014. This indicates that Russia’s economic development tends to decouple from carbon emissions from coal consumption and thus shows little dependence on coal energy and fewer contradictions between its economic growth and environmental conservation.

Similar to Russia, the value of the decoupling elasticity index in terms of year-by-year effects in South Africa also fluctuated, with a trend of SD, END, SD, RD, and SND to RD from 2011 to 2018. However, the decoupling elasticity of the cumulative annual effect consistently showed SD. This indicates that South Africa’s economic development has also decoupled from carbon emissions from coal consumption and is driven more by diversified energy consumption. This again confirms the previous results and shows that although the efficiency of coal use in South Africa needs to be improved, efforts to diversify energy sources have been effective.

### 4.2 Spatial decomposition and decoupling analysis

**Fig 4:**
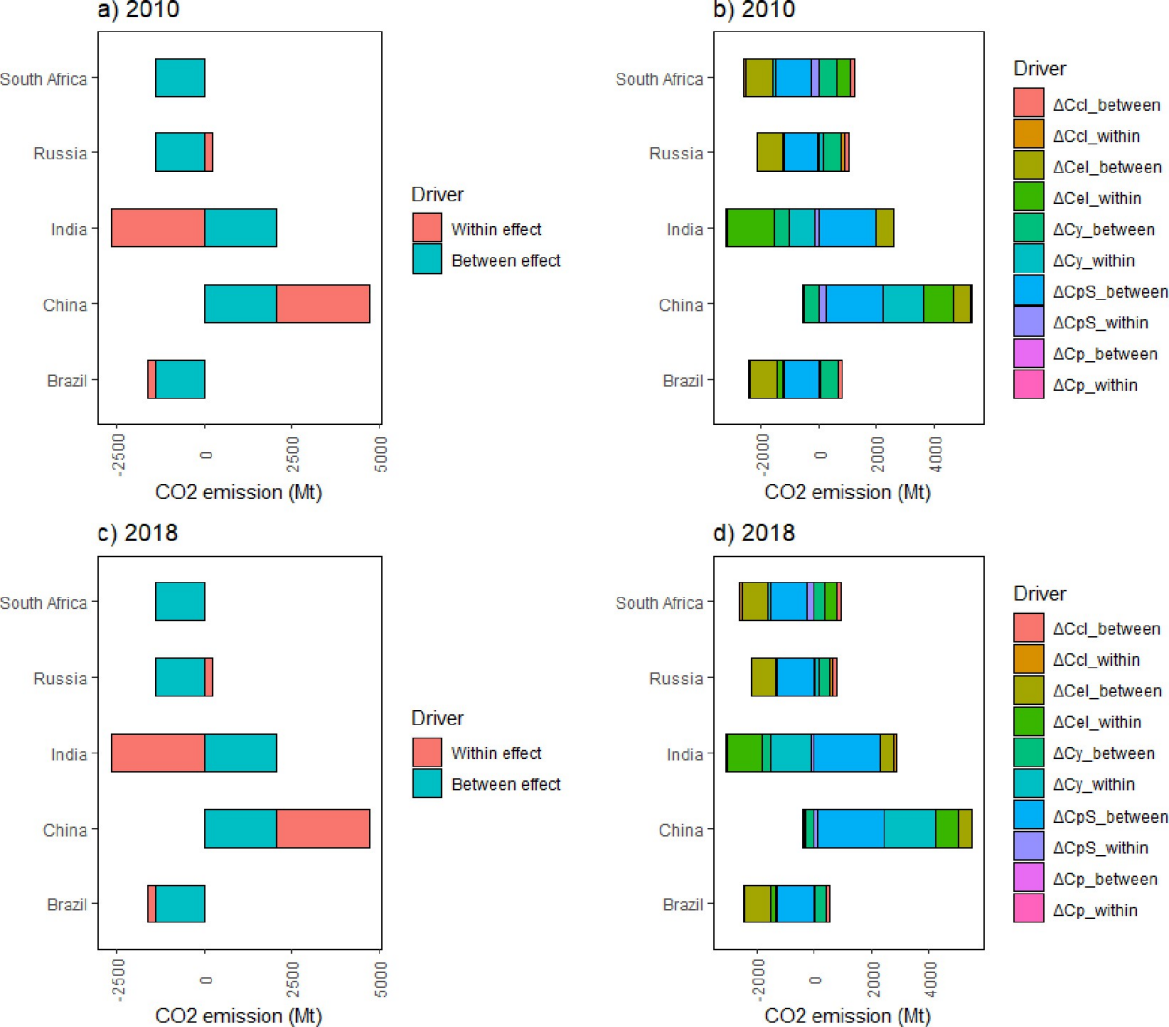
Decomposition of the spatial dimensional drivers of CO_2_ emission from coal consumption in BRICS countries. Note: [1] ΔC_CI_: CO_2_ emission intensity effect; ΔC_EI_: coal consumption intensity effect; ΔC_y_: economic output per capita effect; ΔC_PS_: population structure effect; ΔC_P_: population size effect; [2] “between” means between groups and “within” means within groups.

BRICS countries exhibit different trajectories of carbon emission distributions due to their different economic development levels and resource endowments. To better analyze the difference in spatial regional distribution, this study further conducted spatial decomposition and spatial decoupling analyses, as illustrated in Fig 4.

Overall, the spatial distribution of the two country clusters is consistent in 2010 and 2018, indicating that the distribution of carbon emissions from coal consumption in BRICS countries has not fundamentally changed over time. In terms of intra- and inter-cluster decomposition, both China and India were the biggest contributors to carbon emissions, with China was also the largest contributor to the spatial distribution within its cluster group. Brazil was the smallest contributor to the spatial distribution of CO_2_ emissions, both within and between clusters, followed by South Africa, which had the smallest population size and economic volume. Russia was the main contributor to the variation in spatial distribution within clusters. The spatial differences between the two clusters have continued to widen, mainly because of the increase in CO_2_ emissions in China and India. However, positive changes were also observed. For example, although China is the largest contributor to CO_2_ emissions in terms of both intra- and inter-group decomposition, its energy consumption intensity has a weakening effect on carbon emissions, implying that China has made steady progress in improving its coal utilization technology and coal consumption efficiency. In contrast, India shows more extensive high-emission use of coal. Theoretically, as long as China improves its coal use technology and coal consumption efficiency faster than the other four countries (including India), the overall CO_2_ emissions of BRICS countries will decrease.

From the perspective of decomposition within the group, China’s energy consumption intensity has had a weakening impact on carbon emission compared with India, meaning that India continues to use more coal resources while China continuously restructures its energy mix. This is consistent with previous analyses and the results of existing studies. Thus, India is more reliant on coal consumption among BRICS countries, resulting in more carbon emissions. Coal consumption intensity contributed negatively to all countries in Group I and India in Group II, while it contributed positively to China in Group II. However, all countries’ spatial differences tended to decrease. Economic output per capita contributed positively to all countries in Group I and China in Group II, while it contributed negatively to India in Group II, and both drive wider spatial differences. This suggests that the increase in economic output in all BRICS countries increases carbon emissions and that this spatial difference is increasing due to the different levels of economic development in each country, especially in India. The population structure contributed negatively to all countries in Group I and positively to all countries in Group II, and the distribution of spatial differences narrowed for all countries in Group I and widened for all countries in Group II. This reflects the fact that the energy demand of China and India, as two large populous countries, depends largely on the country’s population and that high-speed urbanization is one of the factors underlying high carbon emissions. Therefore, compared with South Africa, Brazil, and Russia, China and India’s higher energy demands lead to spatial distribution differences in BRICS countries.

**Fig 5:**
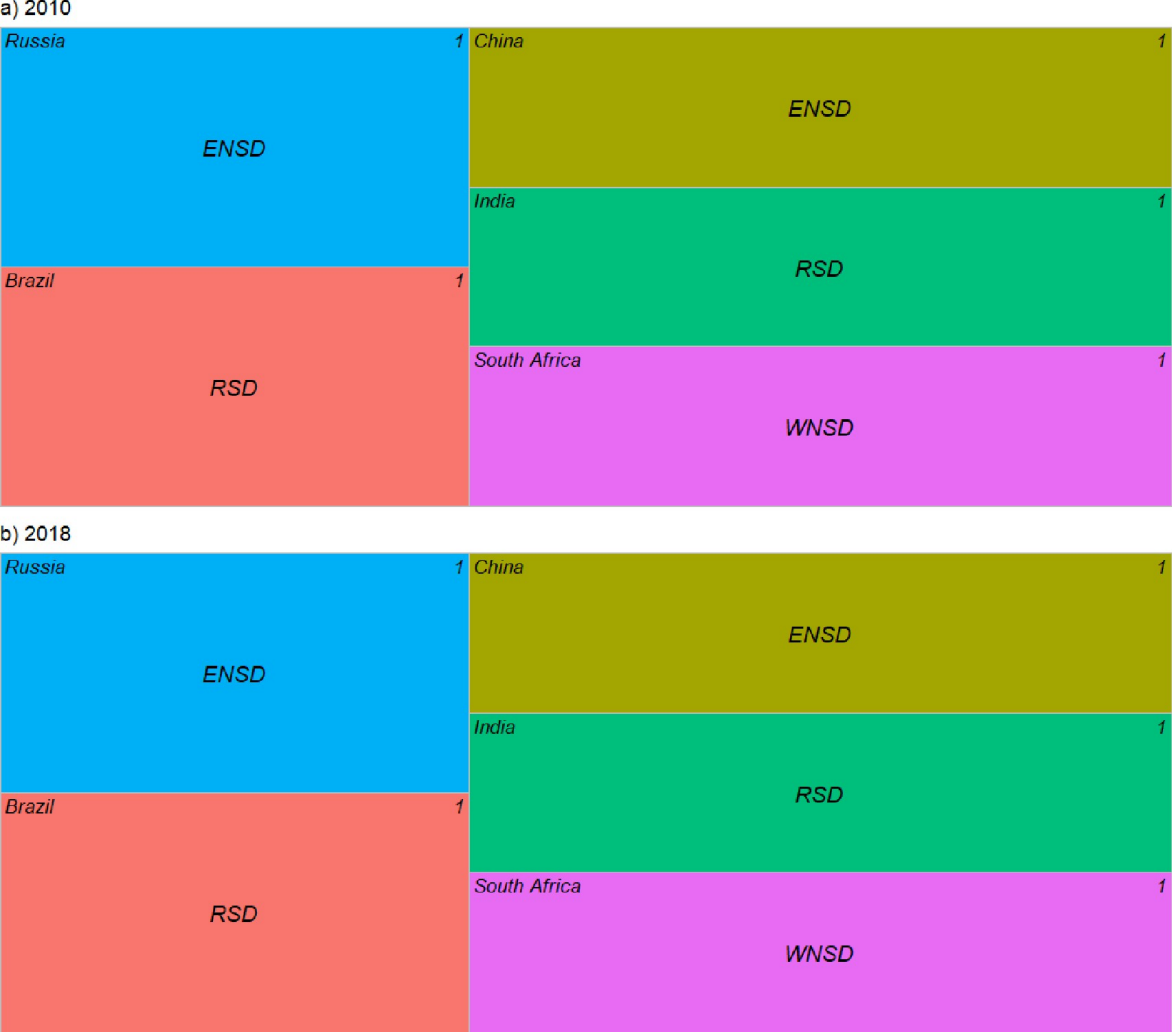
Spatial decoupling of CO_2_ emissions-GDP per capita for coal consumption in BRICS countries (2010–2018). Note: SNSD, strong negative spatial decoupling; WSD, weak spatial decoupling; RSD, recessive spatial decoupling; WNSD, weak negative spatial decoupling; SSD, strong spatial decoupling; ENSD, expansive negative spatial decoupling; RSC, recessive spatial coupling; ESC, expansive spatial coupling.

To research the effect of CO_2_ emissions and economic output per capita on CO_2_ emission changes in different BRICS countries, the spatial decoupling decomposition approach was applied, with the specific decomposition shown in Fig 5.

Overall, carbon emission from coal consumption in the five countries were not decoupled from economic output in either 2010 or 2018, with Brazil and India showing recessive spatial decoupling (RSD), Russia and China showing expansive negative spatial decoupling (ENSD), and South Africa showing weak negative spatial decoupling(WNSD), which is a common state in developing countries that must maintain fast economic development along with intensive industrialization to solve social problems, including poverty [53]. The effect of economic output per capita on the differences in the spatial distribution of carbon emissions within their respective groups is negative for Russia and China and positive for the rest of the countries, indicating that Russia and China are the main contributors.

**Fig 6:**
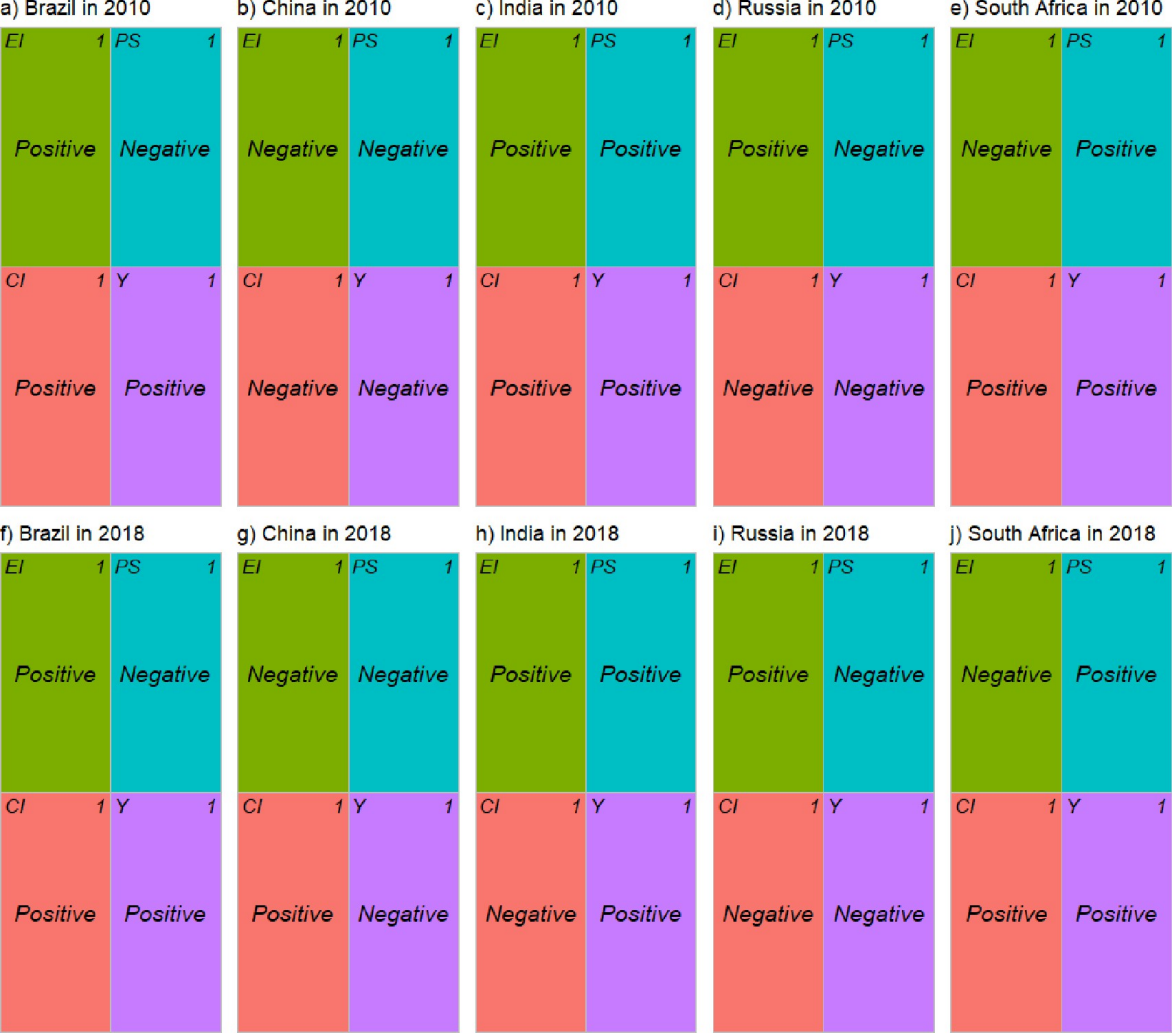
Results of decomposing the distribution effect of CO_2_ emissions from coal consumption in the BRICS subgroups (2010–2018). Note: EI = energy consumption intensity; CI = carbon emission intensity; PS = population structure; Y = GDP per capita.

Fig 6 shows the results of decomposing the distribution effect of CO_2_ emissions from coal consumption in BRICS subgroups from 2010 to 2018. Different country clusters clearly exhibited different degrees to which drivers contributed to the carbon emissions-GDP per capita distribution effect. CO_2_ emission intensity, coal consumption intensity, economic output per capita, and population structure all had positive or negative effects on the distributional effect of the carbon emission-economic output per capita association in BRICS countries, indicating that each BRICS country has country-level heterogeneity in how the four factors influence the distributional effect of CO_2_ emissions. The heterogeneity may be partly due to country-specific characteristics (e.g., economic development, population size, and resource endowment). For instance, the impact of CO_2_ emission intensity on spatial decoupling varies in India and China and had a positive and negative impact in India and China in 2010 and a negative and positive effect on these countries in 2018, respectively. This may be relevant to the fact that China began to increasingly use advanced coal technologies and renewable energy sources, while India had not yet shifted away from its high dependence on coal energy (Chen et al., 2018). Thus, the CO_2_ emission intensity has contributed to narrowing the gap in the spatial distribution of CO_2_ emissions within the group for China but widening the gap for India.

## 5. Conclusions and policy implications

Emerging economies are playing an increasingly vital role in the international community and have become an important driving force of global economic development. Simultaneously, these emerging economies, represented by BRICS, are also the main contributors to global CO_2_ emissions increases. Among BRICS, China, India, and South Africa are characterized by a significantly high-carbon energy structure. If these three countries fail to adjust their energy structures, BRICS countries will remain the main contributors to global carbon dioxide emissions.

Based on the results obtained here, we argue that China is systematically promoting a low-carbon green energy transition away from its dependence on coal consumption, based on its energy endowment and the “Double Carbon” strategy. Moreover, its CO_2_ emission reduction process has demonstrated China’s ability to meet its climate change commitments. Although China is still the biggest user of coal worldwide, it will be able to achieve its CO_2_ emission reduction target if it can steadily replace traditional coal energy with green energy, actively develop low-carbon and zero-carbon industries, and promote a smooth transition towards a low-carbon energy structure. India is under great pressure to develop its economy and improve people’s livelihood. Its CO_2_ emission reduction process indicates that boosting economic growth is a priority; hence, an energy structure dominated by coal consumption will be maintained for a long time, and CO_2_ emissions will continue to increase. In view of India’s important role in global CO_2_ emission reduction, international cooperation and supervision should be strengthened under the Paris Agreement and other UN climate change conferences to clarify India’s obligations to combat climate change and fulfill its commitments. The international community should also do its best to provide India with the financial and technical support needed to implement advanced energy-saving and carbon dioxide reduction technologies, accelerate its energy transformation and industrial restructuring, popularize renewable energy, improve electricity and energy structure and efficiency, and avoid increased coal consumption. Regarding South Africa, which has unique geographical advantages for the development of renewable energy, such as wind and solar energy, the government has attached importance to combating climate change and maintaining cooperation with the international community. In addition, South Africa should actively seek financial and technical assistance from the international community, promote the development of renewable energy in accordance with the planning goals, reduce the proportion of coal consumption and its reliance on coal energy, and promote the transition towards low-carbon energy. In Brazil and Russia, coal is not the primary source in their energy structures, although as signatories to the Paris Agreement, they should fulfill their commitments to develop low-carbon and zero-carbon industries, actively promote energy transformation, and contribute to global CO_2_ emission reduction. In conclusion, BRICS countries should fully exploit appropriate and available mechanisms to develop their economies while also fulfilling their commitments to combat climate change, reduce CO_2_ emissions, and make positive contributions to achieving the climate goals of the Paris Agreement.

Climate change is a common concern worldwide, and a series of climate change agreements, including the Paris Agreement, are driving the world towards a new energy era characterized by decarbonization. However, turbulent international conditions hinders this process. Unilateralism, military conflicts, and energy crises have forced many countries to continue consuming coal. As an important factor that contributed to starting and propelling the industrial revolution and moving mankind from an agricultural society to an industrial society, coal is still the second largest source of energy for human society. Thus, global de-coalization will represent a long-term and arduous task.
